# Distributed Acoustic Sensing for Seismic Monitoring of The Near Surface: A Traffic-Noise Interferometry Case Study

**DOI:** 10.1038/s41598-017-11986-4

**Published:** 2017-09-14

**Authors:** Shan Dou, Nate Lindsey, Anna M. Wagner, Thomas M. Daley, Barry Freifeld, Michelle Robertson, John Peterson, Craig Ulrich, Eileen R. Martin, Jonathan B. Ajo-Franklin

**Affiliations:** 1 Lawrence Berkeley National Laboratory, Energy Geosciences Division, Berkeley, 94720 USA; 2 University of California, Berkeley, Department of Earth and Planetary Science, Berkeley, 94720 USA; 3 U.S. Army Cold Regions Research & Engineering Laboratory (CRREL), Alaska Research Office Fairbanks, Ft, Wainwright, 99703 USA; 4 Stanford University, Institute for Computational & Mathematical Engineering (ICME), Stanford, 94305 USA

## Abstract

Ambient-noise-based seismic monitoring of the near surface often has limited spatiotemporal resolutions because dense seismic arrays are rarely sufficiently affordable for such applications. In recent years, however, distributed acoustic sensing (DAS) techniques have emerged to transform telecommunication fiber-optic cables into dense seismic arrays that are cost effective. With DAS enabling both high sensor counts (“large N”) and long-term operations (“large T”), time-lapse imaging of shear-wave velocity (*V*_*S*_) structures is now possible by combining ambient noise interferometry and multichannel analysis of surface waves (MASW). Here we report the first end-to-end study of time-lapse *V*_*S*_ imaging that uses traffic noise continuously recorded on linear DAS arrays over a three-week period. Our results illustrate that for the top 20 meters the *V*_*S*_ models that is well constrained by the data, we obtain time-lapse repeatability of about 2% in the model domain—a threshold that is low enough for observing subtle near-surface changes such as water content variations and permafrost alteration. This study demonstrates the efficacy of near-surface seismic monitoring using DAS-recorded ambient noise.

## Introduction

The Earth’s near surface—the top tens of meters of the subsurface—provides the foundation that supports our modern infrastructure. Changes to the near surface can lead to hazardous conditions. For example, ground subsidence caused by permafrost thaw can damage buildings; subsurface dissolution processes can lead to devastating sinkholes. Because many such changes can manifest themselves as time-lapse variations in velocity and/or attenuation of seismic waves, seismic monitoring has the potential to provide early warning of near-surface hazards.

In order to provide warnings before failures occur, an effective near-surface seismic monitoring system needs to utilize measurements that have sufficient resolution and extent, both spatially and temporally. This in turn requires deployment and continuous operation of dense sensor arrays, which unfortunately is rarely feasible with conventional sensors (e.g., geophone) because long-term costs are prohibitively high. With the advent of fiber-optic distributed acoustic sensing (DAS) techniques, low-cost, low-maintenance dense arrays are feasible at kilometer scales and beyond^1^. As a result, DAS offers new opportunities for seismic monitoring of the near surface.

DAS repurposes telecommunication optical fibers as multichannel seismic arrays. In contrast to conventional arrays that consist of spatially-discrete electronic sensors, a DAS system utilizes a single optoelectronic interrogator unit that can sample tens of kilometers of optical fiber at sub-meter channel spacing^1^. The interrogator functions by sending short pulses of laser light into the fiber-optic cable and then derives strain or strain rate signals from the strain-induced optical distortions in Rayleigh backscattered light (scattered by micro-heterogeneities in the glass core of the fiber). Time-for-distance conversion allows strain signals to be recorded at spatially localized regions of the fiber, hence transforming the cable into a densely sampled sensor array.

DAS generates digital waveforms that are familiar to seismic practitioners, but because DAS is a distributed sensor, waveforms obtained at each channel are not a point measurement but are strains measured over a spatial distance. This distance is commonly referred to as gauge length, and one must not confuse gauge length with channel spacing: whereas channel spacing can be as small as 25 cm (as it only needs to be longer than the spatial duration of the laser pulse^2^), gauge length needs to be long enough (typically ≥8 meters^3^) to ensure optimal signal-to-noise ratio (SNR). The effect of gauge length is equivalent to applying a moving average filter to the spatial axis of strain measurements that have a sampling interval of channel spacing. In this way, although the spatial resolution of a stand-alone DAS channel is close to the gauge length, the spatial resolution of a DAS array is intermediate between the channel spacing and the gauge length.

For near-surface monitoring, DAS could be an enabling technology particularly for ambient-noise-based approaches^4^. Ambient seismic-noise methods utilize the ubiquitous vibrations generated by natural or anthropogenic sources such as wind, rivers, and vehicles. By cross-correlating ambient noise recorded by a receiver pair, we can retrieve coherent waves that travel from one receiver (acting as a virtual source) to the other without using active or passive seismic sources (e.g., earthquakes, explosives). The resulting ambient-noise interferometry^5, 6^ approaches are particularly useful for seismic monitoring^4^ since they do not require repeated source deployment. Nevertheless, because sensors amenable for monitoring are often sparsely distributed, information retrievable from a typical noise-based monitoring system can rarely extend beyond apparent-velocity perturbations along paths of a few station pairs (often referred to as *dV/V* measurements^7, 8^). This allows the detection of changes along the paths, but not where on the paths the changes are occurring. With the dense spatial sampling provided by DAS, spatial distributions of near-surface changes can be better resolved through time-lapse imaging.

In particular, DAS allows time-lapse imaging of shear-wave velocity (*V*_*S*_) structures using methods such as multichannel analysis of surface waves^9^ (MASW). Although surface waves often dominate virtual wavefields that are generated with ambient-noise interferometry, multichannel arrays rarely are available for continuous recordings of ambient noise. Consequently, time-lapse *V*_*S*_ imaging studies often use surface waves that are acquired from periodically repeated active-source surveys^10, 11^. The limitations of this approach are twofold: (1) Great care must be taken in the surveys to ensure repeatability; (2) Temporal resolution of the monitoring is limited (dictated by the acquisition interval). With DAS, multichannel recordings of surface waves can be continuously retrieved with ambient-noise interferometry, hence making noise-based approach applicable to time-lapse *V*_*S*_ imaging.

In this paper, we investigate the utility of linear DAS arrays for time-lapse *V*_*S*_ imaging with ambient noise. Using traffic noise recorded on a 110-meter-long DAS array oriented perpendicular to a nearby road, we retrieve surface waves that are suitable for *V*_*S*_ imaging and are sufficiently stable for near-surface monitoring when stacked over 24 hours. With the stacked surface waves, we then generate ~500 sets (24 hour rolling window with 1 hour interval for three weeks of continuous recording) of time-lapse *V*_*S*_ profiles via multimodal inversion of surface waves. Because no discernible near-surface changes had occurred during the three-week monitoring period, we use the variations of the *V*_*S*_ profiles to estimate the time-lapse repeatability of near-surface monitoring when facing realistic data errors and inversion non-uniqueness. Our analysis shows a *V*_*S*_ repeatability of about 2% in the top 20 meters of the profiles that are well constrained by the data. This makes DAS-recorded ambient noise suitable for monitoring near-surface changes such as water content variations and permafrost thaw.

## Field deployment and data acquisition

### Field site and DAS array layout

**Figure 1:**
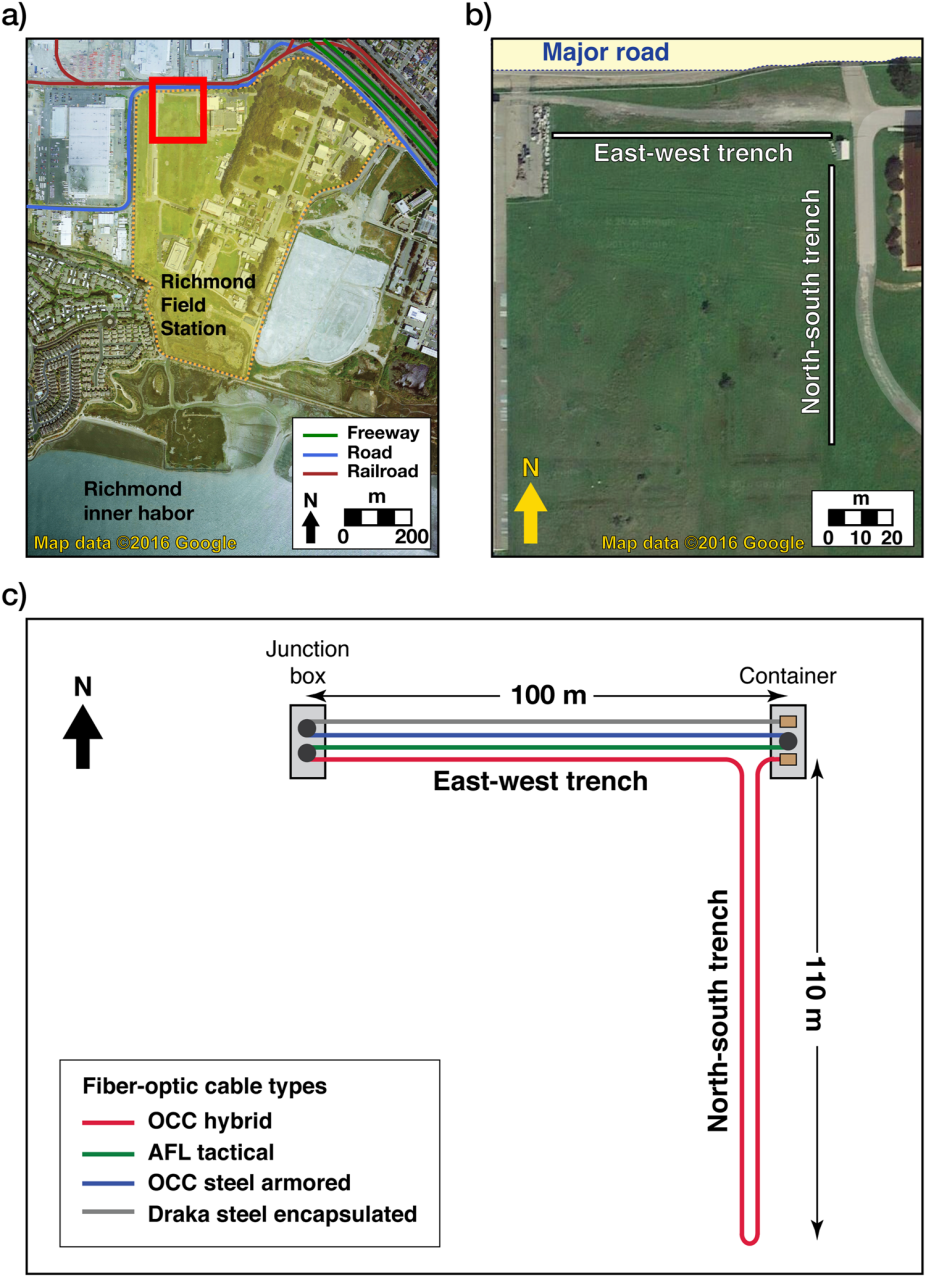
Field site location and layout of the DAS array. (**a**) Field site location (denoted by red rectangle) shown over an aerial map of the Richmond Field Station. (**b**) Zoom-in view of the field site and the L-shaped DAS array. (**c**) Schematic of fiber-optic cable layout. Maps in (**a**) and (**b**) were retrieved from https://goo.gl/j8Av4S. “Richmond Field Station, CA” Map (20 Apr. 2016). Google Maps. Google.

Our field test was conducted at the Richmond Field Station (RFS), a University of California, Berkeley facility located on the San Francisco Bay. We installed an L-shaped trenched DAS array near the RFS boundary next to an east-west oriented road (Fig. 1a,b). The east-west leg of the array (~100 m) is largely parallel to the road and the north-south leg (~110 m) is mostly perpendicular to the road. The minimum distance between DAS channels and the nearest road edge is ~25–30 m. At a slightly greater distance, noise is generated by an infrequently utilized rail corridor; the site is also several hundred meters distant from a major interstate highway. Because of the close distance, vehicle traffic on the boundary road is the predominant source of noise to the array.

The DAS array was installed in surface trenches emplaced with an automated trencher. The trenches were 50 cm deep. The north-south trench was narrow (10 cm) as only one fiber-optic cable was placed in there. The east-west trench was made wider (46 cm) to accommodate four fiber-optic cables of different packaging but identical glass fiber (Corning fiber); these cables were laid side by side for examining if cable packaging can significantly affect DAS sensitivity. All fibers were subsequently spliced in series and buried with the excavated soil to ensure DAS-ground coupling. Fiber bends and spliced connection points were carefully managed to limit optical losses. One end of the fiber was passed into a container office, where it was connected to a DAS system (Silixa iDAS^TM^; Elstree, UK). Gauge length of the system was fixed at 10 meters for the entire survey.

### Fiber-optic cable selection

For investigating the influence of cable packaging on DAS sensitivity, the four cables under comparisons are: (1) a gel-filled, polyethylene-coated hybrid cable containing both single-mode and multi-mode fibers manufactured by Optical Cable Corporation (OCC); (2) a tight-buffered, polyethylene-coated tactical cable manufactured by AFL Telecommunications LLC; (3) a gel-filled, steel-armored cable manufactured by OCC; (4) a tight-buffered, steel-tubing encapsulated cable manufactured by Draka. For simplicity, we use shorthand OCC hybrid, AFL tactical, OCC armored, and Draka encapsulated to denote these cables. In each cable, one strand of single-mode fiber was used for DAS acquisition.

### Data acquisition

Over a three-week monitoring period (April 4–26, 2015), DAS data were continuously acquired at 1 kHz temporal sampling and 1 meter channel spacing. The L-shaped DAS array generated a 2.7 terabyte ambient-noise dataset that was stored as ~31,000 individual files, each containing a 1 minute of noise recording. The total number of DAS channels (excluding bent portions of the cables at turning points) is ~97 in the road-perpendicular (north-south) array and ~94 in the road-parallel (east-west) array.

## Data characteristics

### Cable sensitivity comparison

**Figure 2:**
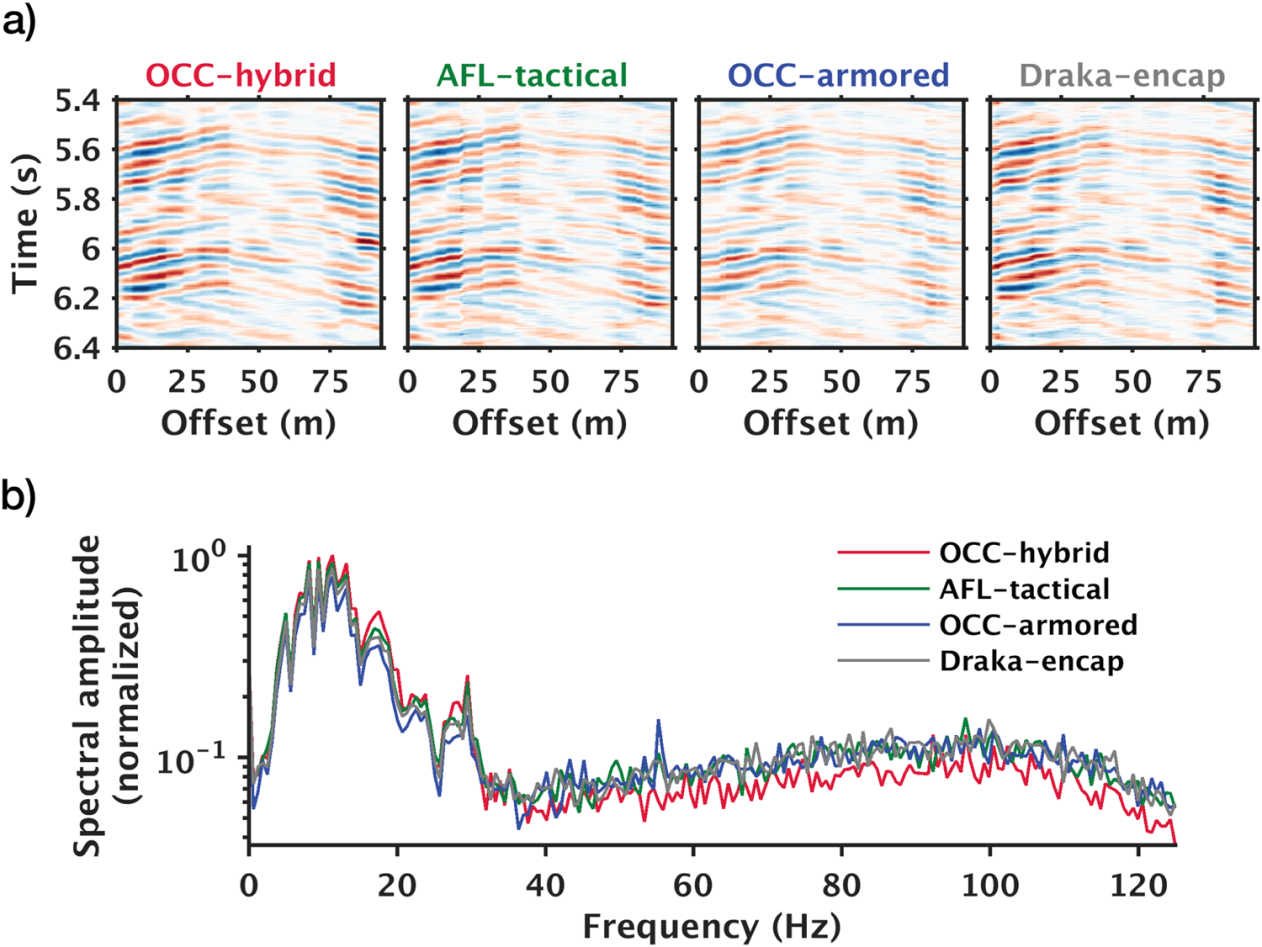
Comparisons of identical traffic noise recorded by fiber-optic cables of different packaging. (**a**) Zoom-in view of 1-second noise records. (**b**) Mean spectral amplitudes computed using identical spatial and temporal windows (spatial window = offset range as in (**a**); temporal window = 1 minute).

We first compare sensitivities of the four cables in the road-parallel array (Fig. 2). Despite the different packaging, all four cables have similar spectral sensitivity in the dominant frequency band of traffic noise (0.6–37 Hz) (Fig. 2b). From this observation, we arrive at the same conclusion as an earlier study^12^, mainly that the influence of cable packaging on DAS sensitivity is minor for traditional straight single-mode cables. Hence when deciding cable packaging for DAS that are used in traffic-noise interferometry, cost, durability, and ease of installation should be the main considerations.

### Influences of array orientations

The road-perpendicular array receives primarily traffic noise that propagates along the axis of the fiber, whereas the road-parallel array receives mostly broadside waves (refer to Figs S1 and S2 in Supplementary Information for more detailed explanations). For ambient-noise interferometry with a linear array, we aim to retrieve coherent waves that travel along the axial direction of the array, and to do so, the dominance of noise propagating along the fiber axis is preferable. For this reason, we focus primarily on the processing and analysis of the data acquired on the road-perpendicular array.

## Methods

**Figure 3:**
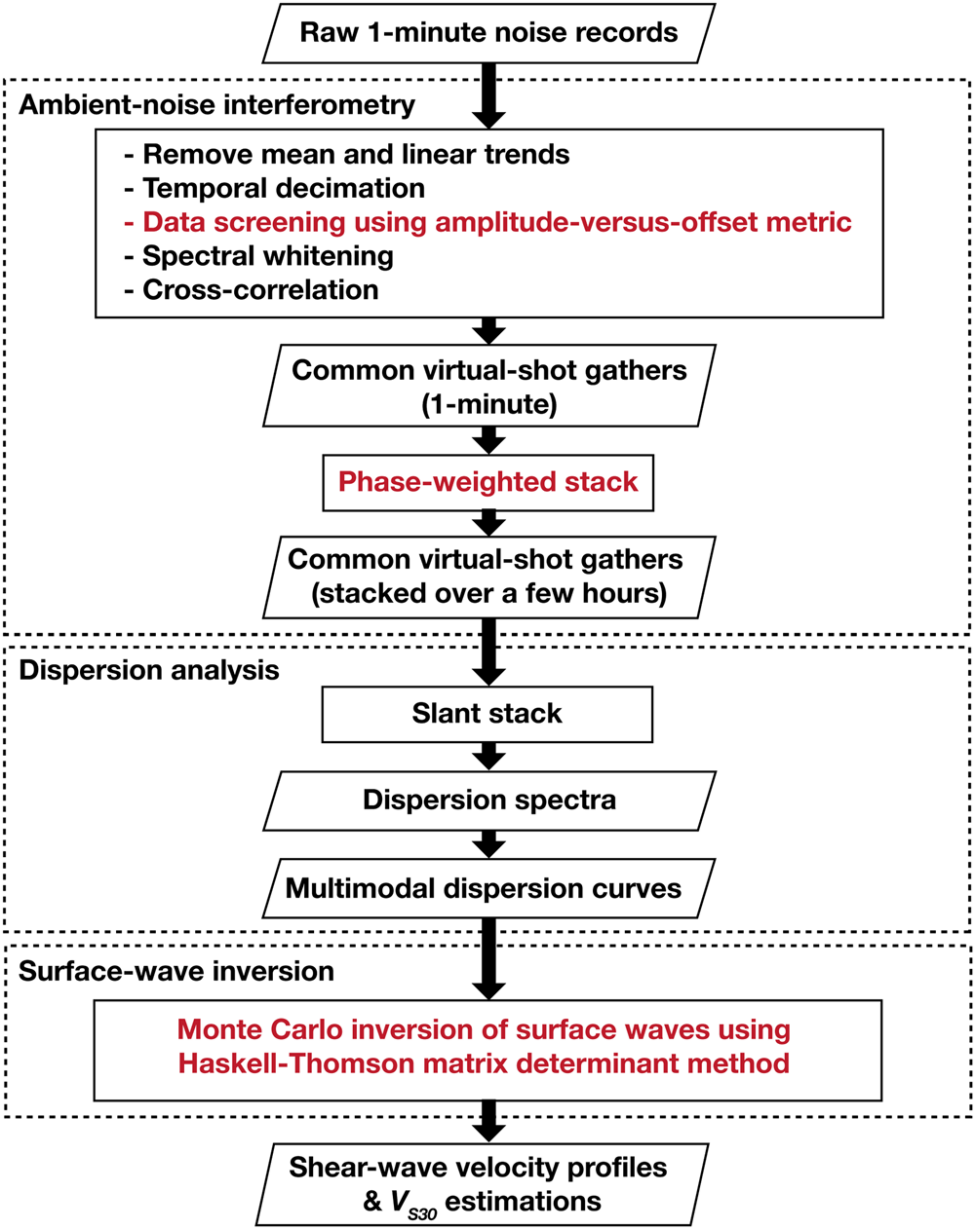
Workflow illustration that starts from raw noise records and ends with shear-wave velocity profiles. Black fonts describe commonly-used steps. Red fonts describe unusual steps taken to improve results.

Figure 3 is a summary of the complete workflow which starts with raw noise records and ends with inverted *V*_*S*_ profiles. Most of the steps required for converting noise records into common virtual-shot gathers are similar to established noise-correlation procedures^13^, but we have omitted temporal normalization (as it degrades the quality of the virtual gathers) and added a data-screening step prior to spectral whitening. For the subsequent cross-correlation, the channel furthest from the road was treated as the virtual source. Noise recorded on the virtual-source channel was then cross-correlated with recordings of the other channels to generate common virtual-shot gathers. Next, we apply phase-weighted vertical stacking^14^ to the virtual shot gathers to improve both the signal-to-noise ratio (SNR) and the temporal stability. Finally, we use the post-stack gathers for dispersion analyses and *V*_*S*_ inversion. In this section, we will describe steps involved in data screening, stacking, and surface-wave inversion; descriptions of other conventional steps are easily accessible in ambient-noise literature and thus are not repeated here (relevant details are provided in the Supplementary Information).

### Data screening with an amplitude-versus-offset metric

The dominance of waves that propagate along the axial direction of the array is necessary for retrieving high-quality surface waves. This requires the presence of near-array vehicles in each of the 1-minute time slices used for noise correlations. We approximate these vehicles collectively as an off-end point source. When the source is present, we expect a power-law decrease in wave amplitudes as distance from the source increases (largely due to geometrical and scattering loss). This allows us to devise a data-screening procedure that examines the scaling between the root-mean-square (RMS) amplitudes of the noise records and the offsets relative to the DAS channel closest to the road. Details about the data-screening procedure and its effects can be found in the Supplementary Information.

### Stacking

Vertical stacking of the common virtual-shot gathers increases SNR of the post-stack data by suppressing incoherent signals. Because of such suppression, stacking homogenizes fluctuations caused by spatiotemporal variations in noise sources and thus stabilizes the data. This in turn improves seismic repeatability (a measure of data similarity in the absence of discernible subsurface changes), an indispensable metric for verifying the causal relationship between subsurface changes and seismic variations.

**Figure 4:**
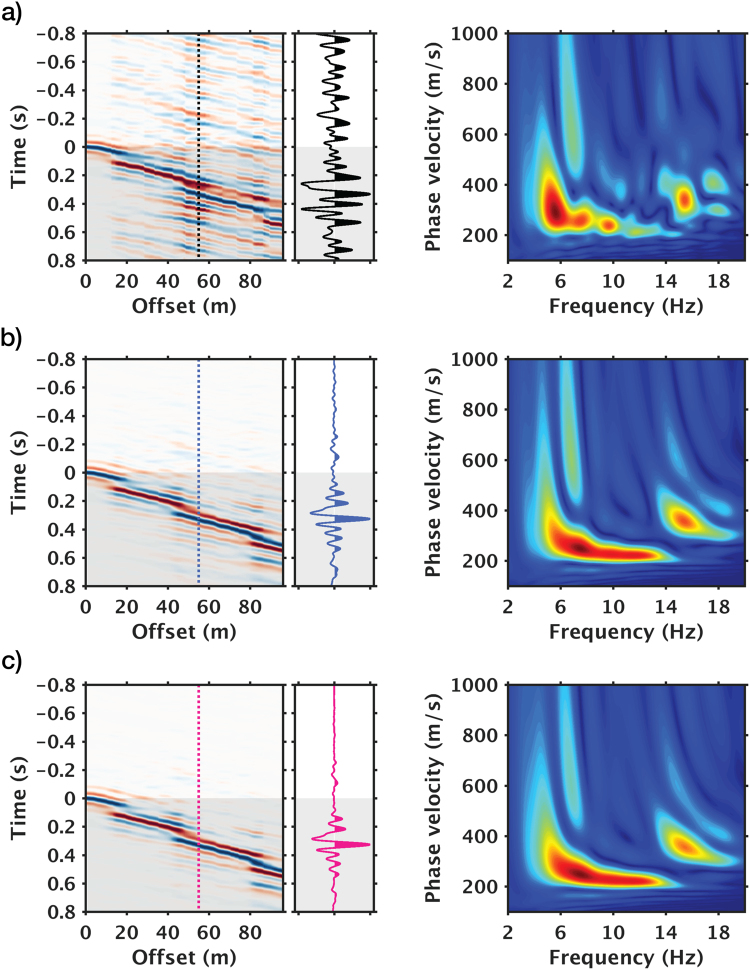
Comparisons between mean stack and phase-weighted stack of common virtual-shot gathers. Left: Time-offset displays and close-up view of a single trace (shaded areas denote causal portions of the virtual-shot gathers); right: corresponding dispersion spectra in velocity-frequency domain. (**a**) Pre-stack gather from 1-minute noise-correlation. (**b**) 1-hour stack via mean stacking. (**c**) 1-hour stack via phase-weighted stacking. Dotted lines in time-offset displays the location of the trace shown in the close-up view.

Increasing stack count improves data quality, but for time-lapse monitoring, also reduces the temporal resolution at which we can observe subsurface changes. To balance the trade-off between data quality and temporal resolution, we use phase-weighted stacking (PWS) to achieve high SNR with fewer records than what would be needed with a mean stack (Fig. 4); we also look for the shortest epoch duration sufficient for stabilizing the data stacks.

**Figure 5:**
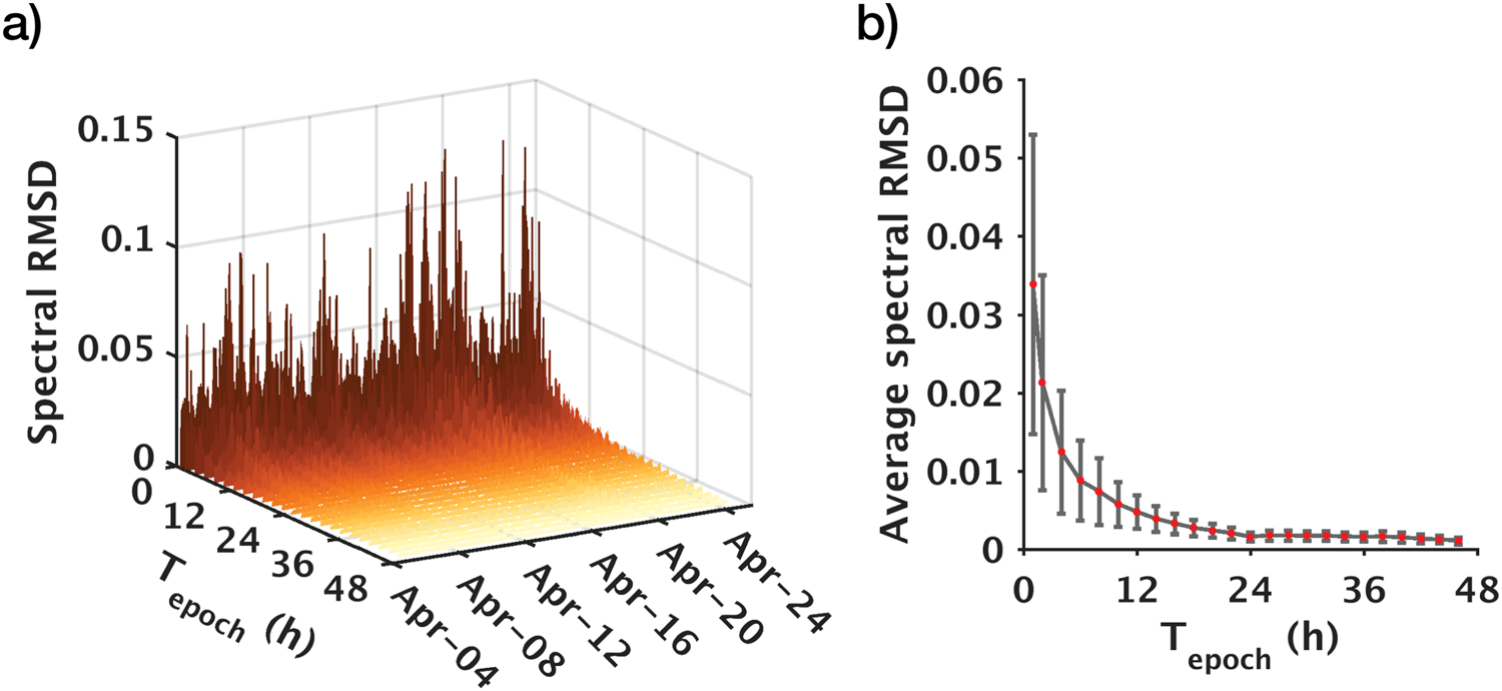
Relationship between stacking time of a single epoch and time-lapse repeatability. (**a**) Expanded view of the three-week monitoring period. (**b**) Collapsed view averaged over the three-week monitoring period. T_epoch_ = Stacking time of a single epoch in hours; spectral RMSD = root-mean-square deviation of dispersion spectra relative to the baseline (the first epoch). In (**a**), color gradation from dark to light denote stacking time from short to long. In (**b**), red dots and grey error bars denote mean and standard deviation, respectively.

We measure data stability using the root-mean-square deviation (RMSD) of the dispersion spectra for all epochs relative to the first epoch (Supplementary equation S2). Since dispersion curves are the form of data that are used to invert for subsurface properties, RMSD evaluation applied to the dispersion domain is a more appropriate measure of stability than time-domain metrics typically used in reflection-based monitoring. Figure 5 shows that longer epoch durations generally yield higher stability, but the rate of improvement diminishes after 24 hours. We therefore conclude that an epoch duration of 24 hours best balances the trade-off between data quality and temporal sampling (For steps involved in PWS, refer to Supplementary Information).

### Surface-wave inversion

**Figure 6:**
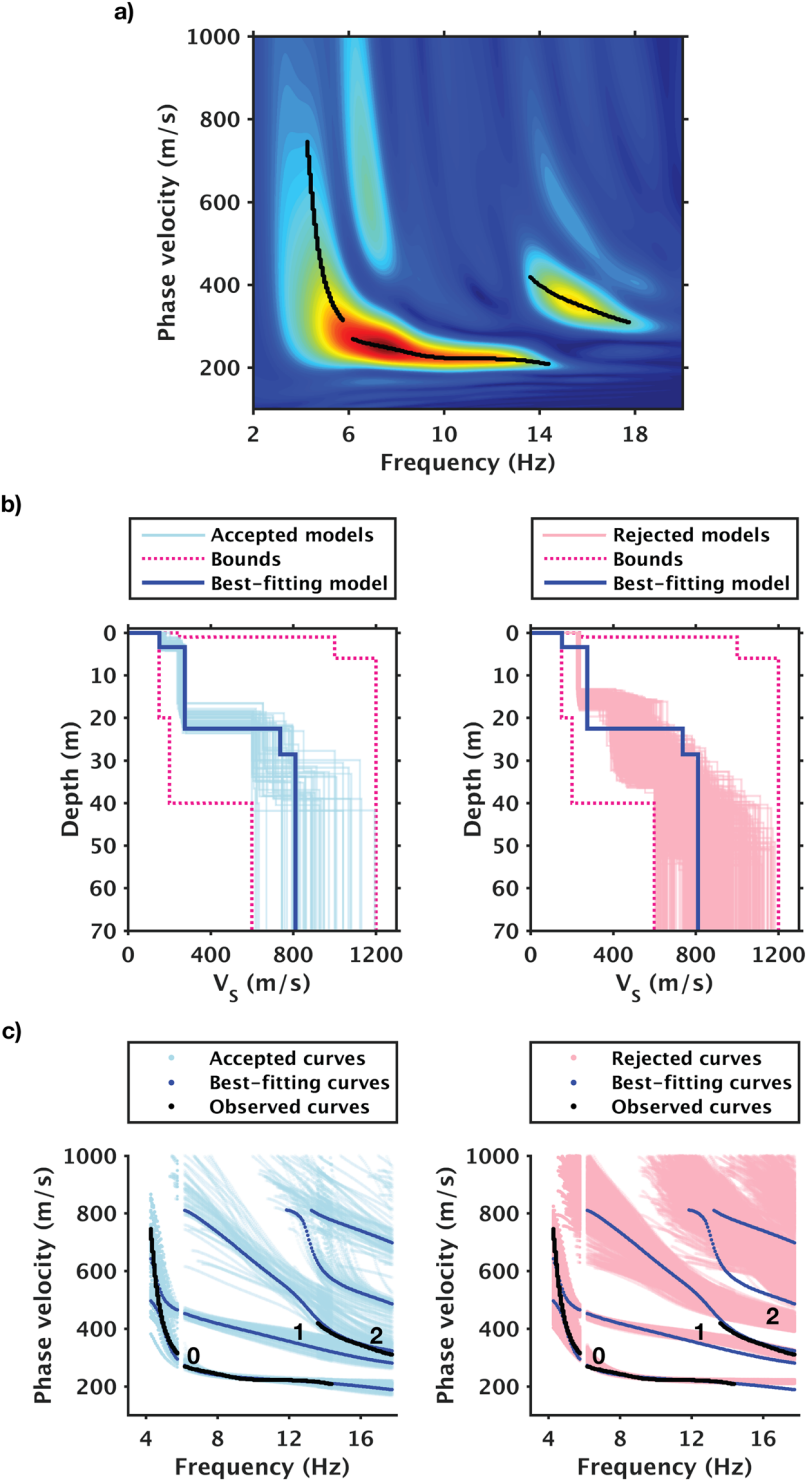
Dispersion measurement and inversion results of a 24-hour stack. (**a**) Dispersion measurements. (**b**) Top 0.1% best-fit models. (**c**) Observed and model-predicted (using profiles shown in (**b**)) dispersion curves. Black dots in (**a**) denote observed multimodal dispersion curves; 0, 1, and 2 in (**c**) denote fundamental mode, 1^st^ higher mode, and 2^nd^ higher mode. Left and right columns in (**b**) and (**c**) show accepted and rejected models respectively.

We use dispersion curves that were retrieved from 24-hour stacks to invert for *V*_*S*_ structures (Fig. 6a). In these stacks, the presence of higher modes is obvious, but mode-number assignments are not straightforward. We use Haskell-Thomson matrix determinant method for the inversion^15^ (hereafter abbreviated as the determinant method), as it allows higher modes to be accounted for without mode-number assignments. The method solves the inverse problem by searching for models that can minimize the determinant of a model-predicted propagator matrix (i.e., the Haskell-Thomson matrix) whose frequency and velocity terms are replaced with the observed dispersion curves. In this way, the determinant method compresses the two-step procedure of the conventional approach that first treats these terms as unknowns to be solved for (by equating the determinant to zero) and then look for models that can minimize the differences between these solutions and the observed dispersion curves. Because this method does not require the time-consuming step of root finding, it is computationally efficient while avoiding the error-prone step of mode assignment.

Due to nonlinearity, the inverse problem is better solved using global search methods. For each epoch, we use a Monte Carlo (MC) random sampling of 5.5 × 10^5^ velocity profiles to search for the best -fit model^16^. For each epoch, the inversion takes only ~2.5 minutes on 10 cores (2.3 GHz Intel Xeon processors). For details regarding the inversion parameters, refer to Table S1 in Supplementary Information.

### Data Availability

All data supporting this study are accessible upon request. Please contact the corresponding author to request access: Dr. Jonathan Ajo-Franklin (JBAjo-Franklin@lbl.gov).

## Results

### Inversion results of a single epoch

Figure 6 shows a single-epoch example of the dispersion data and the top 0.1% best-fit *V*_*S*_ profiles obtained from the inversion. Because solutions obtained with the determinant method alone could be susceptible to incorrect local minima^16^ despite global sampling, we use dispersion curves for further screening of these best-fit models. We compute the model-predicted dispersion curves, compare them against the observed dispersion curve, and reject models that do not provide satisfying data fits. Because this secondary constraint is only applied to the best MC solutions, it is computationally low cost. In our case, the accepted and rejected profiles are distinguished based upon the misfit between the observed high-frequency mode branch (13–18 Hz) and the 2^nd^ higher mode in model predictions (Fig. 6c).

The inversion results illustrate the presence of the “mode kissing” phenomenon^17^, and relatedly, the merits of the determinant method. “Mode kissing” occurs when portions of higher modes appear to have merged with the fundamental mode. It often causes mode misidentification and the subsequent biases in the estimated shear-wave velocities. Because the determinant method does not require mode identification, the inversion is not biased from the start and is able to automatically distinguish the fundamental mode from the higher modes (1^st^ higher mode in our example).

### Inversion results for all epochs

**Figure 7:**
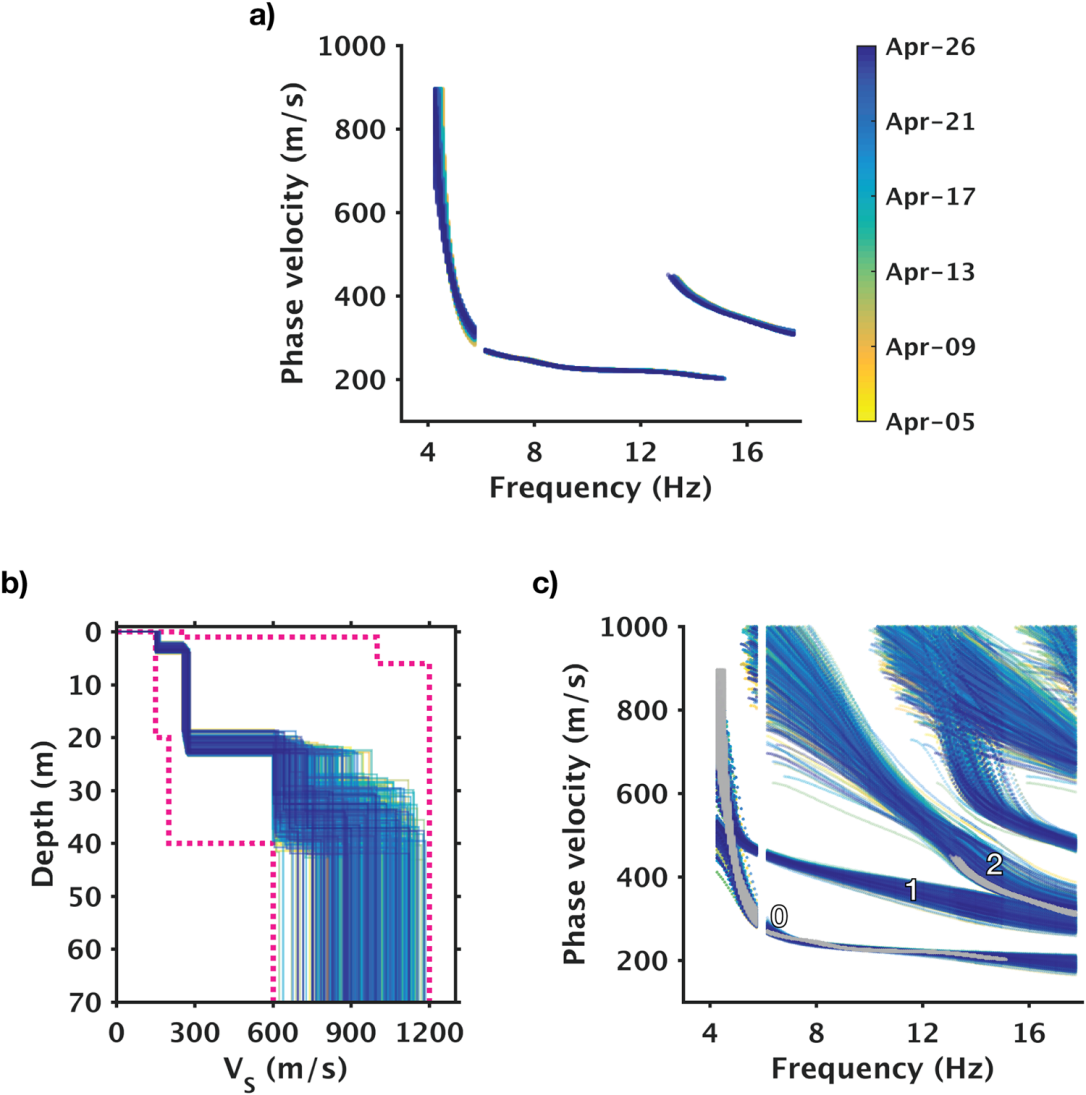
Dispersion curves and inversion results of all 502 epochs. (**a**) Observed dispersion curves. (**b**) Topmost best-fit shear-wave velocity profiles. (**c**) Comparisons of observed and model-predicted dispersion curves. Color coding in all three panels is identical to color bar shown in (**a**). In (**c**), grey dots denote observed dispersion curves (same as in (**a**), but all displayed in grey); rest of color-coded dots denote the corresponding model prediction; 0, 1, and 2 denote fundamental mode, 1^st^ higher mode, and 2^nd^ higher mode.

Over the three-week monitoring period, we use a 24-hour rolling window with 1-hour interval to construct 502 epochs of time-lapse measurements (each epoch is a 24-hour stack) for *V*_*S*_ inversion. Because no systematic changes are present in the virtual gathers, the corresponding dispersion curves can be viewed as repeated measurements that carry realistic data variations (Fig. 7a). By examining how repeatable the associated *V*_*S*_ profiles are, we can then assess the accuracy of the *V*_*S*_ estimates, and correspondingly, the lower limits of time-lapse *V*_*S*_ changes that can be reliably resolved.

### Inversion accuracy and time-lapse resolution

The accuracy of MC inversion is affected by the following issues:

- *Solution convergence*: If the solutions have not converged, a stochastic method like MC may return very different solutions for similar measurements.
- *Non-uniqueness*: Surface-wave inversion is a mix-determined problem, given that shallower structures are sampled by waves of all frequencies whereas deeper structures are only sampled by low-frequency waves^18^. Solution non-uniqueness is inevitable for model parameters that are not well constrained by the data.
- *Stability*: If an inverse problem is unstable, small perturbations in data could lead to large changes in the solution^19^.

The ensemble of the best-fit models (Fig. 7b) show consistent estimates at depths above 20 meters, suggesting that the solutions have converged and the inverse problem is stable. Models become noticeably variable at depths below 20 meters, indicating that information provided by the observed dispersion curves is not enough to fully constrain this part of the model. Nevertheless, the strong velocity contrast at ~20 m depth appears to be a robust feature; it also is the cause of the above-mentioned “mode kissing” phenomenon.

**Figure 8:**
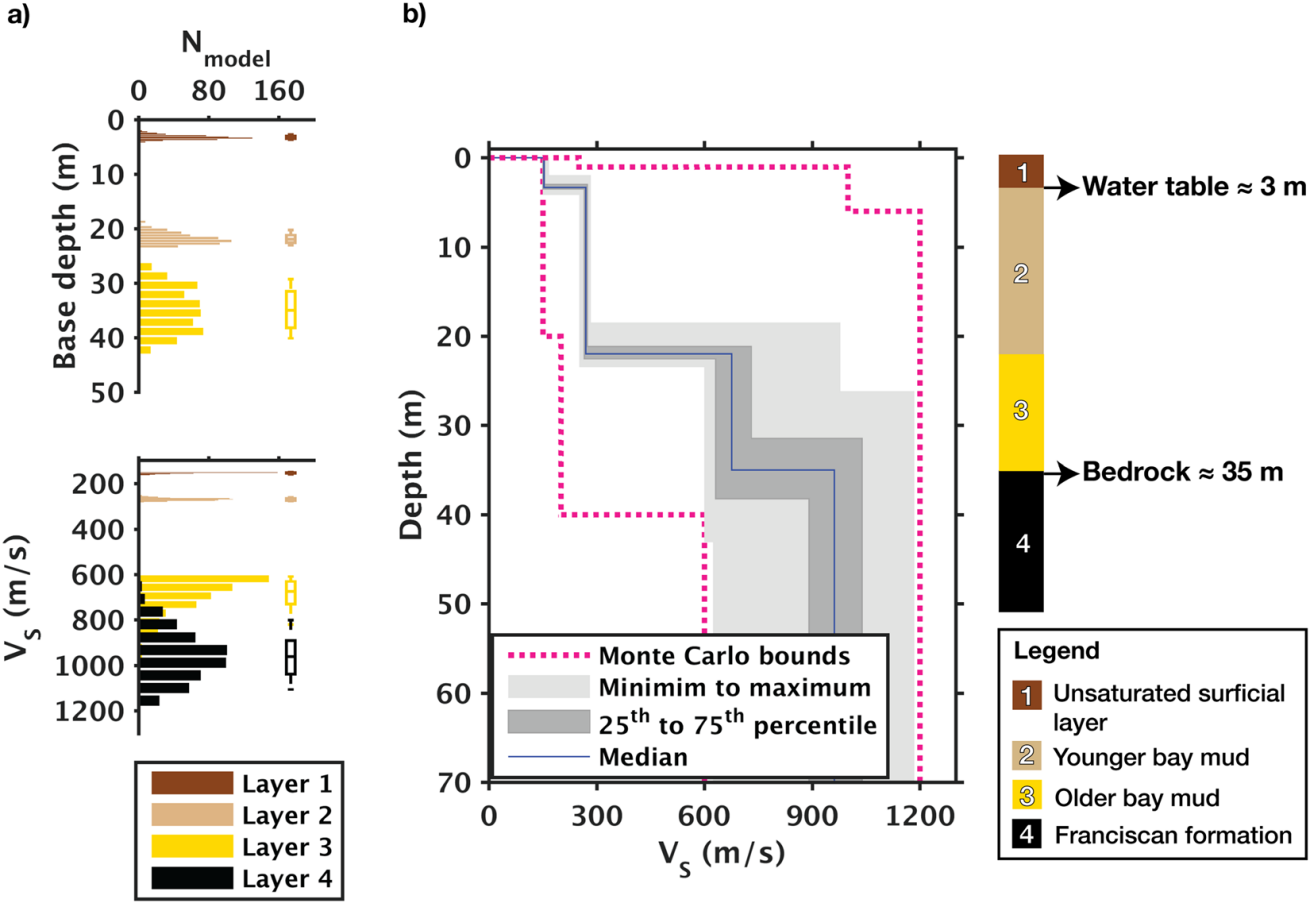
Error analysis and geologic interpretation of the best-fit *V*_*S*_ profiles. (**a**) Histogram and box plots illustrating distributions of base depth and *V*_*S*_ in each layer. (**b**) Error bounds, median *V*_*S*_ profile, and corresponding geologic features.

We view our model-domain repeatability as the lower limit of time-lapse *V*_*S*_ changes that can be reliably resolved. To estimate repeatability in the model domain, we use the median of the model ensemble as the reference value and the 25^th^ and 75^th^ percentiles as the lower and upper bounds; we then treat the deviations of the bounds relative to the references as the model-domain error. Figure 8a illustrates the distributions of the model parameters and the corresponding box plots (the associated numerals are summarized in Table 1). The error analysis indicates that for the top 20 meters of the profile that are well constrained by the data, *V*_*S*_ exhibits less than 2% of variations relative to the reference model. Hence, *V*_*s*_ changes occurring within the 0–20 meter depth range should be reliably resolvable if they are greater than 2%.

**Table 1:** Error estimates of the best-fit models.

Layer	*V*_*S*_ (m/s)		Base depth (m)	
	median	25^th^ percentile–median	Median	25^th^ percentile–median
		75^th^ percentile–median		75^th^ percentile–median
1	153.3	−2.0 (−1.3%)	3.3	−0.3 (−8.5%)
		+2.5 (+1.6%)		+0.2 (+6.7%)
2	270.0	−3.5 (−1.3%)	21.9	−0.8 (−3.4%)
		+4.3 (+1.5%)		+0.6 (+2.9%)
3	675.1	−43.9 (−6.5%)	35.0	−3.4 (−9.9%)
		+56.3 (+8.3%)		+3.2 (+9.2%)
4	961.9	−70.5 (−7.3%)	∞	
		+77.0 (+8.0%)		

### Geologic interpretation

Figure 8b illustrates the median *V*_*S*_ profile and the associated error bounds. Among the three layer interfaces within the profile, the shallowest (~3 m) and the deepest (~35 m) have depths that are consistent with the mean water table and bedrock depths of the site, as measured by prior vertical seismic profiling surveys^20^ and geotechnical studies; the intermediate interface (~20 m) likely marks the transition from the younger bay mud (very soft to medium stiff) to the older bay mud (stiff to very stiff)^21^.

### *V*_*S30*_ estimates

**Figure 9:**
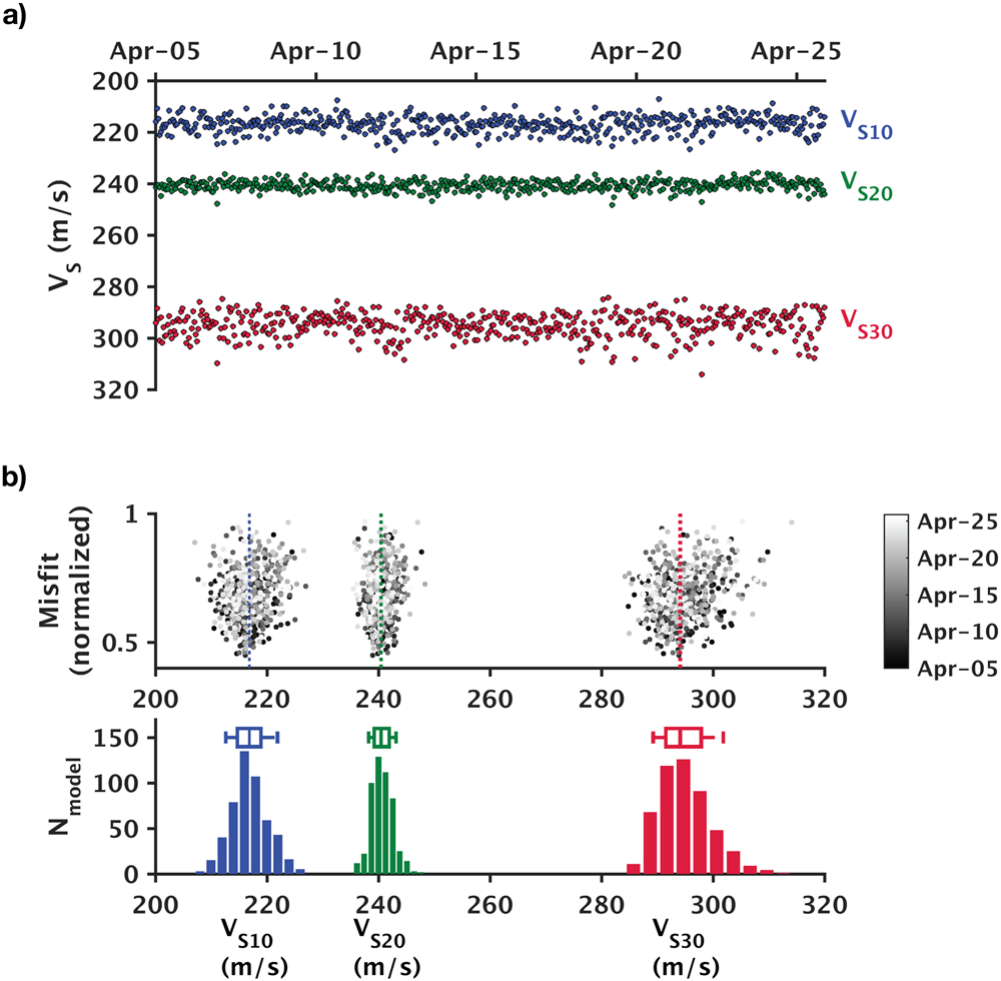
Average shear-wave velocities (*V*_*S10*_, *V*_*S20*_, and *V*_*S30*_) estimated from best-fit models of all 502 epochs. (**a**) Average *V*_*S*_ displayed as functions of calendar time. (**b**) Distributions of *V*_*S10*_, *V*_*S20*_, and *V*_*S30*_ (lower) and corresponding misfits (upper).

*V*_*S30*_, the average shear-wave travel-time of the top 30 meters (Supplementary equation S3), is widely used as a first-order indicator of seismic site conditions^22, 23^. Because surface waves usually are well suited to sampling the top 30 meters, estimating *V*_*S30*_ has been among the main motivations for conducting surface-wave surveys^24–26^. Here we examine the repeatability of *V*_*S30*_, V_*S20*_, and *V*_*S10*_ estimates using the ensemble of best-fit models (Fig. 9b). Table 2 summaries the results of error analyses based upon the median and the 25^th^ and 75^th^ percentiles (similar to Table 1). The errors of all three average *V*_*S*_ estimates do not exceed 1.5% throughout the three-week monitoring period, which again demonstrates the accuracy of the inversion. We can also see that the extent of spread in *V*_*S30*_, *V*_*S20*_, and *V*_*S10*_ correlate closely to the frequency-band limits of the common virtual-shot gathers: Because the wavefield is energetic in the 5–13 Hz range but weak (or absent) at both lower and higher frequencies, *V*_*S10*_ and *V*_*S30*_ are subjected to larger errors and thus show wider spread than *V*_*S20*_. The *V*_*S30*_ range (291.5–297.9 m/s) places our field site in class D (stiff soil; *V*_*S30*_ = 180–360 m/s) according to the International Building Code standards (IBC 2006; Table S2 in Supplementary Information), which is consistent with results of previous studies^27, 28^.

**Table 2:** Error estimates of *V*_*S10*_, *V*_*S20*_, and *V*_*S30*_.

	Median	25^th^ percentile–median	75^th^ percentile–median
*V*_*S10*_ (m/s)	216.7	−2.0 (−0.9%)	+2.2 (+1.0%)
*V*_*S20*_ (m/s)	240.5	−1.2 (−0.5%)	+1.5 (+0.6%)
*V*_*S30*_ (m/s)	294.1	−2.6 (−0.9%)	+3.8 (+1.3%)

## Discussion

Our study illustrates that with DAS, multichannel recordings of surface waves suitable for time-lapse *V*_*S*_ imaging can be obtained via ambient-noise interferometry. And with continuous acquisition of repeatable surface-wave data, imaging uncertainties can be quantitatively assessed. Our imaging results show that for the top 20 meters of the *V*_*S*_ profiles that are adequately sampled by surface waves, we can achieve a time-lapse repeatability of about 2% in the model domain (in terms of Δ*V*_*S*_/*V*_*S*_). Although the resolvable depth ranges will be site-specific (depending on the local ambient-noise source field), we expect comparable time-lapse repeatability levels for the well-constrained portions of *V*_*S*_ profiles at other locations. According to values reported in literature, the 2% limit could easily be exceeded by a variety of near-surface *V*_*S*_ changes caused by processes such as water content variations (up to 10–15%^10, 29^) and freeze-thaw transitions of unconsolidated permafrost (up to 70–80%^30^). Therefore, time-lapse *V*_*S*_ imaging enabled by DAS can be a powerful tool for seismic monitoring of the near surface. Examples of likely applications include monitoring water-table fluctuations in irrigated agriculture and thermokarst early warning for cold-region infrastructure.

In practice, one must also consider the spatial distributions of noise sources. When conditions allow, array geometries that facilitate the retrieval of inter-receiver surface waves should be utilized (i.e., surface waves that travel inline between the virtual source and the virtual receivers). For effective design of arrays in directional noise environments, knowledge of the spatial distributions of dominant seismic noise sources should be considered. As an example, our data analyses illustrate that when using traffic noise recorded on linear DAS arrays, the array perpendicular to the road generated higher quality results when processed using classical cross-correlation approaches. For situations where preferred array geometries cannot be achieved, directional corrections will need to be applied to the dispersion measurements, and such methods have been proposed in literature^31–34^. Though not included in this study, investigating the effectiveness of these approaches should be part of future work.

Lastly, despite being a powerful enabler of both “large N” and “large T” seismic sensing, DAS should not be viewed as a replacement of conventional sensing. Instead, it is becoming a part of the “large N” efforts among the seismology communities to record full seismic wavefields and to reduce or eliminate spatial aliasing^35–37^.

## Conclusion

This is the first end-to-end study that uses infrastructure noise recorded on linear DAS arrays for time-lapse *V*_*S*_ imaging. With a 110-meter-long DAS array deployed perpendicular to a nearby road, we were able to acquire multichannel recordings of surface waves via ambient-noise interferometry. We found that an integration time of 24 hours is sufficient to best balance the tradeoffs between time-lapse resolutions and data stabilities at the site. Over three weeks of continuous monitoring, we obtained ~500 sets of *V*_*S*_ profiles using determinant-based multimodal inversion, and in the absence of discernible subsurface changes, we used variations of these *V*_*S*_ profiles to assess time-lapse repeatability in the model domain. Results suggest that for the top 20 meters of the profiles that are well constrained by the data, we can achieve repeatability of about 2% (in terms of Δ*V*_*S*_/*V*_*S*_)—a threshold low enough for observing near-surface changes such as water content variations and permafrost thaw. In short, this study demonstrates the efficacy of near-surface seismic monitoring using DAS-recorded ambient noise. As a low-cost dense array, DAS could be a powerful tool in establishing smarter systems for monitoring the Earth’s near surface.

## Electronic supplementary material

Supplementary Information
