# Supplementary material for: Distributed Acoustic Sensing for Seismic Monitoring of The Near Surface: A Traffic-Noise Interferometry Case Study
Source: Sci Rep. 2017 Sep 14;7:11620. doi: 10.1038/s41598-017-11986-4 (PMC5599533; doi:10.1038/s41598-017-11986-4)
Supplement: Supplementary file 1 — Supplementary Information [file 41598_2017_11986_MOESM1_ESM.pdf]

Supplementary Information for:

Distributed Acoustic Sensing for Seismic Monitoring of The Near Surface: A Traffic-Noise Interferometry Case Study

Shan Dou[1,+], Nate Lindsey[1,2,+], Anna M. Wagner[3], Thomas M. Daley[1], Barry Freifeld[1], Michelle Robertson[1], John Peterson[1], Craig Ulrich[1], Eileen R. Martin[4], Jonathan B. Ajo-Franklin[1,*]

[1]Lawrence Berkeley National Laboratory, Energy Geosciences Division, Berkeley, 94720, USA
[2]University of California, Berkeley, Department of Earth and Planetary Science, Berkeley, 94720, USA
[3]U.S. Army Cold Regions Research & Engineering Laboratory (CRREL), Alaska Research Office Fairbanks, Ft. Wainwright, 99703, USA
[4]Stanford University, Institute for Computational & Mathematical Engineering (ICME), Stanford, 94305, USA
[*]JBAjo-Franklin@lbl.gov
[+]These authors contributed equally to this work

Influences of array orientations

As shown in Figure S1, traffic noise recorded by the road-parallel (east-west) and road-perpendicular (north-south) arrays have distinctly different characteristics. Data from the road-perpendicular array have steeper moveout and near-uniform dip direction in the time-offset domain. In the frequency-wavenumber domain, the spectral peaks mostly reside in one quadrant and lean toward lower apparent velocities (~240 m/s). Data from the road-parallel array constitute series of crisscrossed hyperbolas that have flatter moveout and non-uniform dips in the time-offset domain. The corresponding spectral peaks are present in quadrants of both positive and negative wavenumbers and cover a wide range of apparent velocities (~100–1000 m/s). The spread-out distributions of spectral peaks indicate that waves recorded by the road-parallel array come from a variety of azimuths.

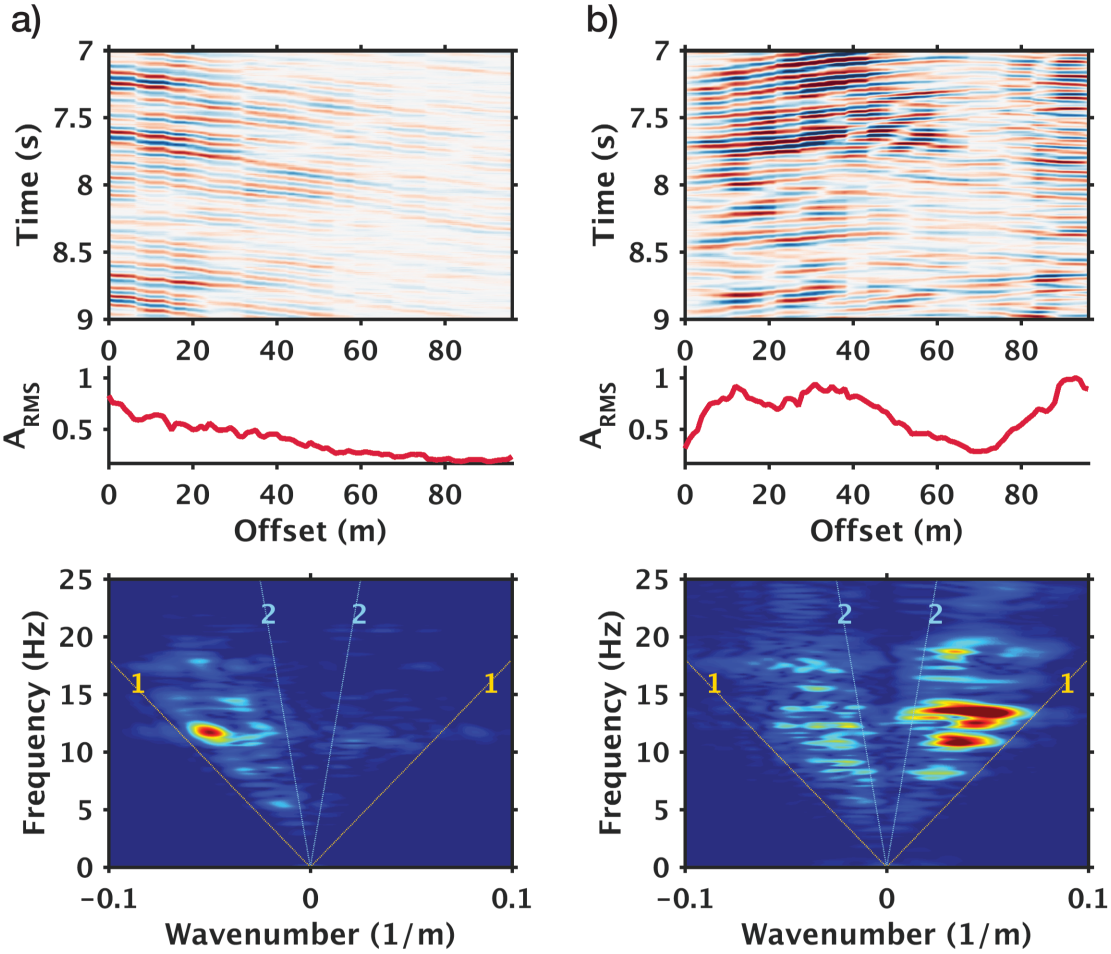

Figure S1. Comparisons of noise records between north-south (road-perpendicular) and east-west (road-parallel) DAS arrays. Upper: time-offset displays; middle: amplitude-versus-offset trends; lower: frequency-wavenumber spectra. (a) North-south array. (b) East-west array. A_{RMS} = root-mean-square (RMS) amplitude computed over 1-minute time-window. In frequency-wavenumber spectra, yellow dotted lines (denoted by 1) denote apparent velocity of 100 m/s; blue dotted lines (denoted by 2) denote apparent velocity of 1000 m/s.

The appearances of noise recordings suggest that the road-parallel array is dominated by broadside waves whereas the road-perpendicular array mainly records waves that travel along the axial direction of the fiber. Such differences result from combined effects of geometric spreading and directional filtering. To provide an intuitive explanation, we illustrate a simple example of P-wave propagation in Figure S2:

Regardless of array orientation, the directional filtering of DAS always leads to a $cos^2\theta$ scaling with respect to waves' incident angle θ (relative to the axial direction of the fiber)[1]. For the road-perpendicular array, geometrical spreading yields an additional scaling of $cos\theta$, which leads to a $cos^3\theta$ response that peaks along the axial direction. As a result, waves that propagate along the axial direction will dominate the DAS recordings. For the road-parallel array, geometrical spreading yields a factor of $sin\theta$. Combined with directional filtering, the resulting response of $sin\theta \cdot cos^2\theta$ reaches zero at the axial direction and maximizes at 35° incident angle. This explains the dominance of broadside waves on the road-parallel array.

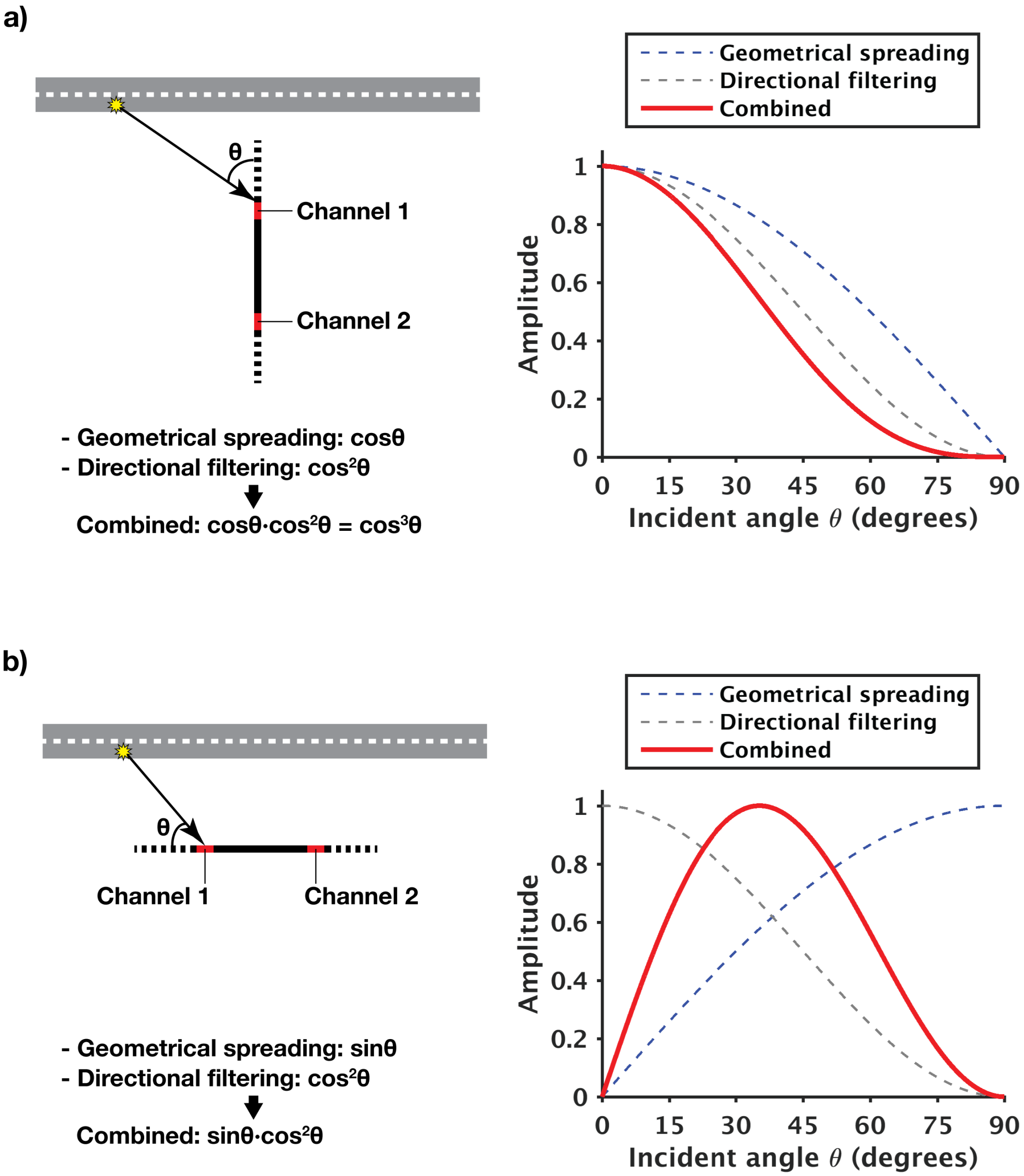

Figure S2. Schematic explanations on influences of DAS array orientations (not to scale). (a) Road-perpendicular array. (b) Road-parallel array. Grey strip denotes road; yellow star denotes noise source (vehicle); arrows denote wave propagation directions; channel 1 and 2 denotes two neighboring DAS channels.

Additional information on data-processing methods

Amplitude-versus-offset metric for data screening

We devise a data-screening procedure based upon the expected power-law scaling between the RMS amplitudes of the noise records ($A_{RMS}^{[i]}$, where i is the channel index) and the offsets relative to the northernmost DAS channel ($x^{[i]}$). We fit the observed $A_{RMS}^{[i]}$ vs. $x^{[i]}$ relationship to a power-law expression $A_{RMS}^{[i]<fit>} = a(x^{[i]})^c + b$, where a, b, and c are curve-fitting coefficients to be solved by minimizing the least-square misfit $\sum_{i=1}^{N}(A_{RMS}^{[i]<fit>} - A_{RMS}^{[i]})^2$. For our dataset, we found that c values close to -2 consistently yield high-quality surface waves. We also incorporate Pearson's correlation coefficient R to evaluate the goodness of fit. The closer R is to 1, the better the fit. Combining these two criteria, we construct a data-screening metric $\zeta = |cR + 2|$. Only when $\zeta < 1$ do we accept a noise record for further processing..

Pearson's correlation coefficient

We use Pearson's correlation coefficient R to assess the goodness of fit. Assuming we have a total of N data points (x_i, y_i) $(i = 1, 2, \cdots, N)$ and we have fitted these data to a curve described by function $y_i^{<fit>} = f(x_i)$, we compute coefficient R via Equation S1 as shown below:

$$R = \sqrt{\frac{SSR}{SSE+SSR}}$$
$$SSR = \sum_i (y_i^{<fit>} - \bar{y})^2 \qquad \text{(S1)}$$
$$SSE = \sum_i (y_i^{<fit>} - y_i)^2$$

where $\bar{y} = \sum_{i=1}^{N} y_i / N$ denotes the mean value of data points y_i; *SSR* denotes sum of squared residuals; *SSE* denotes sum of squared errors. The closer R is to 1, the better the fit.

Effects of screening on data quality

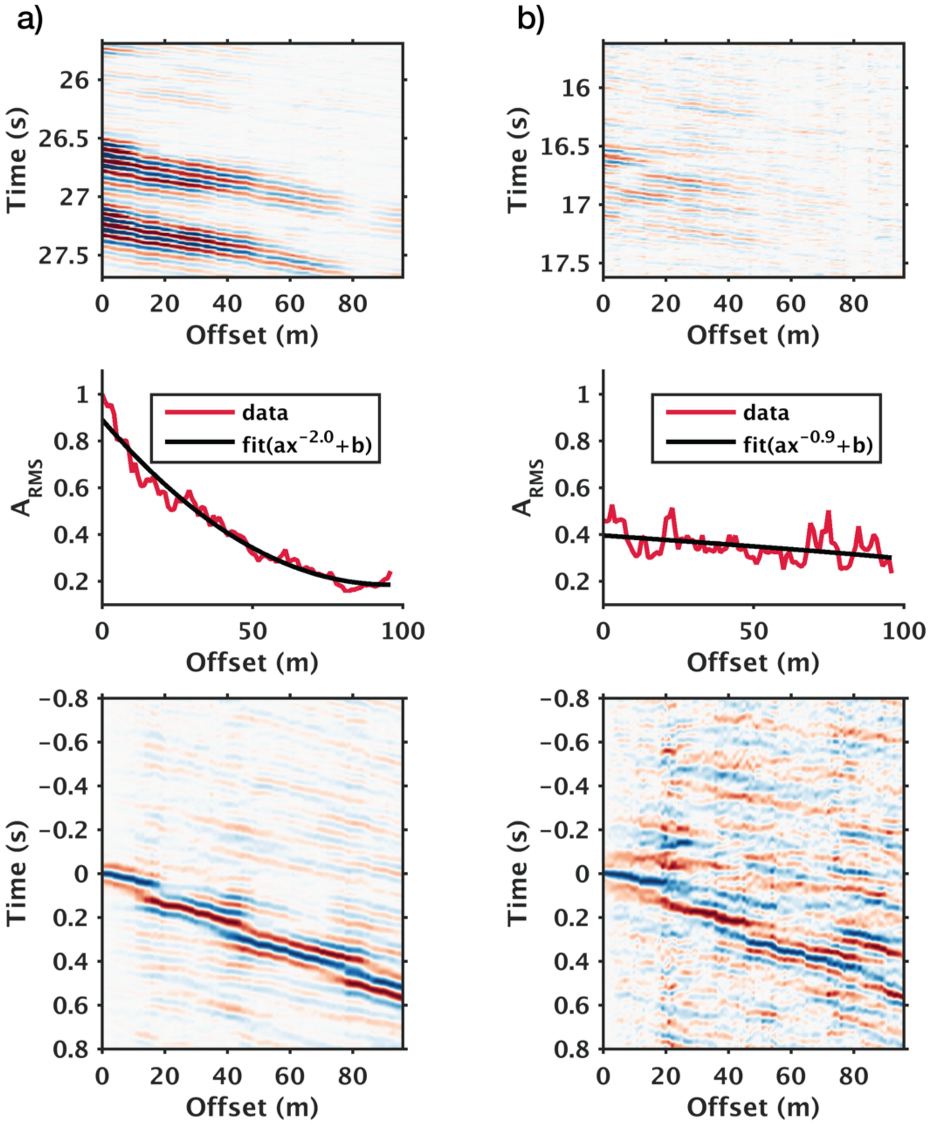

Figure S3. Comparisons of accepted and rejected noise records. Upper: offset-time displays; middle: amplitude-versus-offset trends; lower: common virtual-shot gathers. (a) Accepted noise records. (b) Rejected noise records. A_{RMS} = root-mean-square (RMS) amplitude computed over 1-minute time-window; x = offset in meters; a and b are curve-fitting parameters.

We demonstrate the efficacy of screening on improving data quality in Figure S3. As an example, we compare common virtual-shot gathers obtained from accepted and rejected noise records. The quality of the gather obtained from the accepted noise record ($R = 0.98$; $c = -2$; $\zeta = 0$) is clearly better than its counterpart ($R = 0.43$; $c = -0.9$; $\zeta=1.6$).

Correlation between hourly data rejection rates and traffic pattern

The effectiveness of the data-screening procedure is also confirmed by the correlation between the hourly data rejection rate and the expected traffic pattern. As shown in Figure S4, lower rejection rates correspond to early morning and afternoon-evening rush hours; higher rejection rates correspond to quieter time such as mid- to late-morning, early afternoon, and post-midnight hours. Such correlation is to be expected, as the data-screening metric essentially measures whether or not vehicles are present near the array within each of the 1-minute time window. Therefore the busier the traffic, the lower the rejection rate.

The high rejection rate between 7 am and 8 am is unexpected. Its cause is presently unclear, as we did not conduct independent observations of the traffic during the experiment.

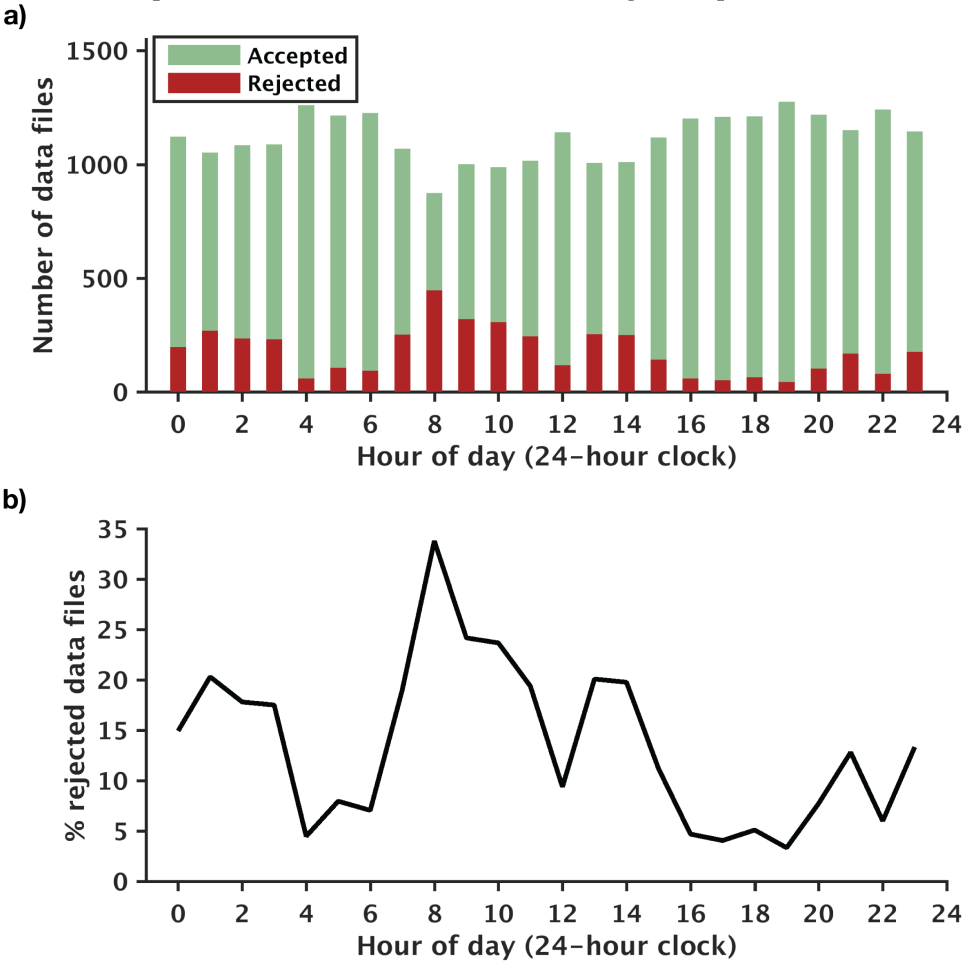

Figure S4. Distributions of hourly data rejection rate. (a) Bar graph illustrating hourly partition between the number of accepted and rejected 1-minute noise records. (b) Hourly rejection rates presented by percentage with respect to total number of 1-minute noise records within each hour. Number of 1-minute noise records within each hour is the cumulative sum computed over the three-week monitoring period.

Slant stack for dispersion analysis

We use slant stack to transform data from time-offset (*t*-*x*) domain to frequency-velocity (*f*-*V*) domain. Starting from a multichannel shot gather consisted of N seismic traces $u(t, x_k)$ (where $k = 1, 2, \cdots, N$ is the trace index), we use the following two steps to transform it into its dispersion spectrum $\widetilde{U}(f, V)$:

- Transform time-domain data into frequency domain via Fourier transform: $\tilde{u}(f, x_k) = \int_{-\infty}^{+\infty} u(t, x_k) e^{-i2\pi f t} dt$.
- Apply offset-dependent phase shift $2\pi f\, x_k/V$ to $\tilde{u}(f, x_k)$, and sum over all the offsets of the shot gather: $\widetilde{U}(f, V) = \sum_{k=1}^{N} \tilde{u}(f, x_k) e^{i2\pi f x_k/V}$.

When looping through a range of plausible phase-velocity values, signals that travel with the same phase velocity as V at frequency f will stack constructively and become part of the spectral peaks in the dispersion spectrum.

Phase-weighted stacking (PWS)

PWS includes three steps[2]:

- Compute instantaneous phase $\Phi(t) = tan^{-1}[\frac{H(s(t))}{s(t)}]$ (in radians), where s(t) is a seismic trace, H(s(t)) is its Hilbert transform.
- Compute mean stack (MS) of both the instantaneous phases $c(t) = \frac{1}{N}\left|\sum_{i=1}^{N} e^{j\Phi_i(t)}\right|$ and the seismic traces $S^{MS}(t) = \frac{1}{N}\sum_{i=1}^{N} s_i(t)$, where j is the imaginary unit; i is the trace index; N is the total stack count.
- Apply phase stack $c(t)$ as a weighting factor to the trace stack: $S^{PWS}(t) = c^{\upsilon}(t)S^{MS}(t)$, where υ is a power term. Larger υ leads to more severe influences from the phase weighting factor ($\upsilon = 1$ in our case).

The weighting factor c(t) is a coherency measure that varies between 0 and 1. When signals are out of phase (incoherent), c(t) is close to 0, causing the corresponding samples in the trace stack to be suppressed. By suppressing incoherent signals, PWS achieves an improved SNR with shorter stacking periods.

Spectral root-mean-square deviation (RMSD) computation

To assess the difference between two dispersion spectra, we compute spectral RMSD using Equation S2:

$$RMSD = \sqrt{\frac{\sum_{i=1}^{N_f}\sum_{j=1}^{N_V}(S_{ij}^{(2)} - S_{ij}^{(1)})^2}{N_f N_V}} \qquad \text{(S2)}$$

where subscript *i* denotes frequency sampling; subscript *j* denotes velocity sampling; N_f is the total number of frequency points; N_V is the total number of velocity points; $S_{ij}^{(1)}$ and $S_{ij}^{(2)}$ denote the two dispersion spectra under comparison.

Model configuration for the inversion

For the inversion, we use a 1D layer-cake model consisting of four layers. Because surface waves are primarily sensitive to shear-wave velocities (V_S), we reduce the number of variables in the inversion by using fixed P- to S-wave velocity ratio (V_P/V_S) and density ρ (V_P/V_S = 1.87, ρ = 2000 kg/m^3). Layer thickness h and shear-wave velocity V_S of each layer are the variables, which leaves a total number of seven variables to be solved by the inversion (h_1, V_{S1}; h_2, V_{S2}; h_3, V_{S3}; V_{S4}). Upper and lower bounds used in the Monte Carlo sampling are shown in Table S1. To further reduce computation time, only normally dispersive models (velocities increase with increasing depths) from the sample pool are used in the inversion. This yields ~0.55 million normally dispersive models, which take up about 37% of the entire sample pool.

Table S1. Upper and lower bounds in Monte Carlo sampling of the inversion variables.

**Layer index**	V_{Smin} **(m/s)**	V_{Smax} **(m/s)**	h_{min} **(m)**	h_{max} **(m)**
1	150	250	1	20
2	200	1000	5	20
3	600	1200	5	20
4	600	1200	-	-

V_{S30} calculation and site classification

V_{S30} is the travel-time average of the shear-wave velocity over the top 30 meters. It is computed via Equation S3:

$$V_{S30} = \frac{30}{\sum_i t_i} = \frac{30}{\sum_i \frac{h_i}{V_{Si}}} \quad \text{(S3)}$$

where *i* denotes layer index included in the top 30 meters; *h* denotes layer thickness; *t* denotes one-way travel time.

Table S2 shows site-condition classes used in building-codes regulations (IBC 2006) and their corresponding V_{S30} ranges. Our estimated V_{S30} range of 284.2–314.1 m/s places the field site in class D (stiff soil), which is consistent with the classification provided by earlier studies[3,4].

Table S2. IBC[*] (2006) site classification with v_{S30} as site[3,4]-condition indicator.

**Site class**	**Soil type description**	**V_{S30} range (m/s)**
A	Hard rock	>1500
B	Rock	760–1500
C	Very dense soil and soft rock	360–760
D	Stiff soil	180–360
E	Soft soil	<180

[*]IBC = the International Building Codes.

References

1 Kuvshinov, B. N. Interaction of helically wound fibre-optic cables with plane seismic waves. *Geophysical Prospecting* **64**, 671-688, doi:10.1111/1365-2478.12303 (2016).

2 Schimmel, M. & Paulssen, H. Noise reduction and detection of weak, coherent signals through phase-weighted stacks. *Geophysical Journal International* **130**, 497-505, doi:10.1111/j.1365-246X.1997.tb05664.x (1997).

3 Wills, C. J. *et al.* A site-conditions map for California based on geology and shear-wave velocity. *Bulletin of the Seismological Society of America* **90**, S187-S208, doi:10.1785/0120000503 (2000).

4 Wald, D. J. & Allen, T. I. Topographic slope as a proxy for seismic site conditions and amplification. *Bulletin of the Seismological Society of America* **97**, 1379-1395, doi:10.1785/0120060267 (2007).
